# Effectiveness of antipsychotics in schizophrenia with comorbid substance use disorder

**DOI:** 10.1192/j.eurpsy.2021.431

**Published:** 2021-08-13

**Authors:** M. Lähteenvuo, J. Luykx, H. Taipale, E. Mittendorfer-Rutz, A. Tanskanen, A. Batalla, J. Tiihonen

**Affiliations:** 1 Dept Of Forensic Psychiatry, University of Eastern Finland, Kuopio, Finland; 2 Department Of Psychiatry, Utrecht University, Utrecht, Netherlands; 3 Department Of Clinical Neuroscience, Karolinska Institutet, Stockholm, Sweden; 4 Psychiatry, University Medical Centre Utrecht, Utrecht, Netherlands

**Keywords:** schizophrénia, antipsychotic, Substance use

## Abstract

**Introduction:**

Schizophrenia is highly comorbid with substance use disorders (SUD), which may negatively impact the course of illness. However, large studies exploring the best lines of treatment for this combination are lacking.

**Objectives:**

We investigated what are the most effective antipsychotics for patients with schizophrenia in preventing the development of substance use disorders and preventing hospitalizations in patients already having substance use disorder.

**Methods:**

We used two independent national cohort registries including all patient with schizophrenia aged under 46 years. Participants were followed during 22 (1996–2017, Finland) and 11 years (2006–2016, Sweden). We studied risk of rehospitalization, and risk of developing an SUD when using vs. not using antipsychotics, using Cox proportional hazards regression analysis models.

**Results:**

45,476 patients with schizophrenia were identified (30,860 in Finland; 14,616 in Sweden). For patients without SUD, clozapine and antipsychotic polytherapy were associated with the lowest risks of developing SUD in both countries. For patients with co-existing SUD, the risk of hospitalization was the lowest during clozapine, polytherapy and long-acting injectable use.

**Conclusions:**

In patients with schizophrenia and comorbid SUD, antipsychotic medications were effective in preventing relapses. In those without an SUD, antipsychotic use was associated with a markedly reduced risk of developing an initial SUD. Clozapine and long-acting injectables should be considered treatments of choice in patients with schizophrenia and SUD, or at risk of developing co-morbid SUD.

**Disclosure:**

ML: Genomi Solutions Ltd, DNE Ltd, Sunovion, Orion Pharma, Janssen-Cilag, Finnish Medical Foundation, Emil Aaltonen Foundation. HT, EMR, AT: Eli Lilly, Janssen–Cilag. JT: Eli Lilly, Janssen-Cilag, Lundbeck, Otsuka.

